# Clinical Evaluation of Patients Using Antineoplastic Chemotherapeutic Agents with Body Composition Monitor Measurement Before and at 3 Months of Treatment

**DOI:** 10.3390/jcm14207148

**Published:** 2025-10-10

**Authors:** Mehmet Turan Ozer, Ertugrul Bayram, Saime Paydas, Semra Paydas

**Affiliations:** 1 Department of Internal Medicine, Faculty of Medicine, Cukurova University, Adana 01250, Turkey; turanozer1@gmail.com; 2 Department of Medical Oncology, Faculty of Medicine, Cukurova University, Adana 01250, Turkey; sepay@cu.edu.tr; 3 Department of Nephrology, Faculty of Medicine, Cukurova University, Adana 01250, Turkey; spaydas@cu.edu.tr

**Keywords:** body composition monitor, overvolemia, antineoplastic chemotherapeutic agent, overhydration

## Abstract

**Background/Objectives**: In patients with malignancy, fluid electrolyte imbalance and renal dysfunction have been demonstrated to increase mortality and morbidity. The objective of this study was to evaluate body composition, and clinical and laboratory tests at baseline and at 3 months during chemotherapy and standard fluid therapy in patients with malignancy. **Methods**: This study included patients with an ECOG performance status of 0–1 who did not have clinically evident organ failure, brain tumors, or a need for intensive care treatment. All received standard fluid therapy and chemotherapy. Examinations, routine laboratory tests, and body composition measurements were performed at the beginning of chemotherapy and again after three months. **Results**: The number of hypervolemic patients increased. Although the body mass index (BMI) did not change compared to the baseline, serum levels of B-type natriuretic peptide (BNP), calcium, albumin, total cholesterol, high-density lipoprotein (HDL) cholesterol, and triglycerides increased at the second measurement. By month three, the frequency of overhydration (OH) increased. There was a significant, positive, moderate correlation between the difference in OH and the difference in BNP (*p* = 0.001). The leukocyte, neutrophil, neutrophil-to-lymphocyte ratio, and neutrophil-to-albumin ratio decreased (*p* < 0.05 for all). Body composition monitor (BCM) measurements revealed that the extracellular fluid/intracellular fluid ratio (E/I) increased at the second measurement (*p* = 0.001). **Conclusions**: The frequency of OH and BNP levels increased at three months. An initial fluid deficit or OH was associated with mortality. OH may mask sarcopenia in patients. Therefore, objective assessment of body composition is important for patient management to avoid OH and predict mortality. However, more studies with larger patient populations and long-term follow-up are needed.

## 1. Introduction

Cancer is defined as the uncontrolled proliferation of cells as a result of damage to some genetic and metabolic pathways. The prevalence of malnutrition in adult cancer patients ranges from 20% to 87%, contingent on the stage, type, or treatment of cancer [1,2].

Cytotoxic chemotherapy agents are prescribed for both curative and palliative purposes, and their dosage is typically determined using the body surface area (BSA) calculation. However, this method does not accurately reflect the patient’s actual body composition [3,4].

Malnutrition and cachexia, which are frequently seen in patients with malignancy, are important prognostic factors that negatively affect response to treatment [5]. These conditions have been shown to increase treatment side effects and negatively affect survival. Early recognition of malnutrition plays a critical role in improving prognosis [5,6].

Fluid–electrolyte balance disorders may develop in cancer patients due to their treatments and may worsen their clinical course by affecting hemodynamic balance. Therefore, accurately and objectively assessing body composition is important for monitoring patients’ clinical status [7]. A body composition monitor (BCM) is a non-invasive device that can determine the amount of fat, muscle, and lean muscle tissue, as well as intracellular and extracellular fluid distribution. It is useful in clinical settings. BCMs are used to assess fluid balance in dialysis patients, intensive care patients, and, more recently, oncology patients [8,9].

In this context, the aim of this study was to evaluate body composition, clinical parameters and laboratory findings in patients with malignancy before chemotherapy and standard fluid therapies and in the third month of treatment to reveal the changes in the treatment process. Monitoring the changes in muscle and adipose tissue as well as fluid distribution will contribute to the directing of patients to individualized supportive treatment approaches [10].

## 2. Materials and Methods

### 2.1. Study Design and Participants

Between 1 June 2023 and 1 December 2023, all 77 patients over the age of 18 who would be treated for the first time in the Medical Oncology chemotherapy unit of Çukurova University Faculty of Medicine, Feyyaz Etiz Oncology-Hematology Hospital were included in the study.

All patients were informed about the study and their written informed consent was obtained. The study was conducted with the approval of the Cukurova University Clinical Research Ethics Committee.

The demographic data, physical examination findings, and routine laboratory test results requested for the purpose of ensuring compliance with chemotherapy were meticulously documented. Body composition analysis was conducted using the BCM (Fresenius Medical Care, Bad Homburg vor der Höhe, Germany) device in accordance with the standard protocols recommended by the manufacturer. The measurements were taken prior to the initiation of chemotherapy and three months following the initial administration. The second measurement, which was independent of the completion time of chemotherapy, was performed at the same time interval for all patients.

In the third-month follow-up, eight patients died. Three patients were transferred to another center due to the earthquake. Three patients could not be reached, and one patient was referred for bone marrow transplantation. Consequently, the third-month measurements were conducted on a total of 62 patients.

### 2.2. Inclusion and Exclusion Criteria

The selection of patients for inclusion in the study was predicated on the fulfillment of specific criteria. Accordingly, patients who agreed to participate in the study and gave informed consent, were 18 years of age or older, had an ECOG performance status of 0–1, did not actively use alcohol, did not have a diagnosis of significant heart failure, impaired consciousness or brain tumor, and did not use drugs or psychoactive substances without physician approval were included in the study. Conversely, patients exhibiting signs of active infection, substantial organ dysfunction, or clinically significant heart failure necessitating intensive care treatment, hyperbilirubinemia or uremia, or who declined to participate in the study were excluded from the study.

### 2.3. Body Composition Monitor Measurements

Body composition measurements were performed with the BCM device (Fresenius Medical Care, Germany). The measurements were performed by the same trained researcher in each patient, in accordance with the manufacturer’s instructions. Prior to the procedure, the patients’ skin surfaces were meticulously cleansed, and the electrodes were strategically positioned on the wrists and ankles of the patients on the same side. Patient information such as height (centimeters), weight (kilograms), gender, and arterial blood pressure (systolic/diastolic, millimeters of mercury) were entered into the device.

**Figure 1:**
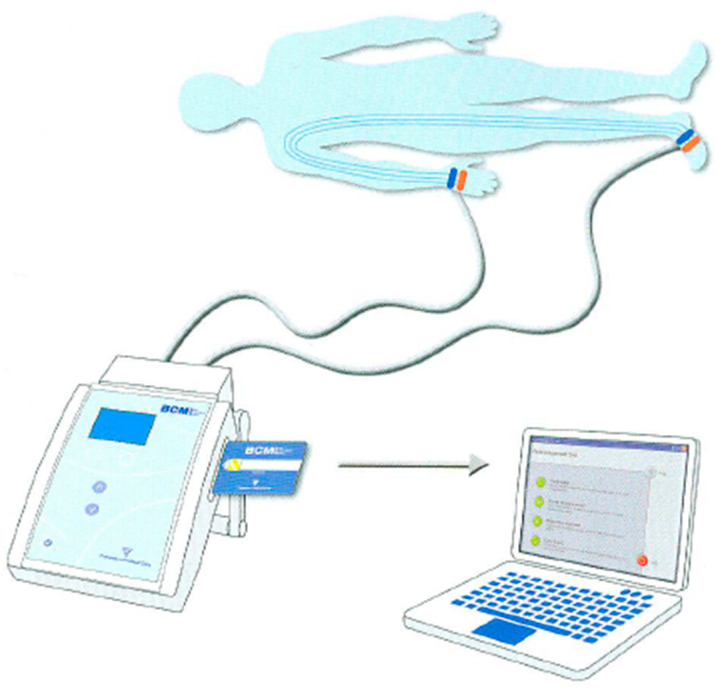
Patient connection diagram and the data transfer process in measurement with the BCM.

During the measurement procedures with BCM, patients were evaluated in a supine position, having fasted and been given a sufficient amount of time to rest. Subsequent to the successful establishment of a connection to the device, the measurement commenced and was concluded within approximately two minutes. The data obtained were recorded on the patient card and transferred to the computer Via the Fluid Management Tool (FMT) of the BCM (Figure 1).

The BCM system is a bioimpedance spectroscopy (BIS)-based apparatus that measures 50 distinct frequencies ranging from 5 kHz to 1000 kHz. It utilizes this data to calculate a variety of physiological parameters, including total body fluid (TBF), intracellular fluid (ICW), extracellular fluid (ECW), lean tissue index (LTI), fat tissue index (FTI), overhydration (OH), and body mass. The measurement time was approximately two minutes and was performed noninvasively.

Overhydration (OH) was defined according to the manufacturer’s validated BCM thresholds as an absolute fluid overload > 1.1 L. Patients were further classified as normovolemic, hypervolemic, or hypovolemic based on extracellular water (ECW) and intracellular water (ICW) distributions. These cut-offs were consistent with previously published studies validating BCM against isotope dilution and clinical fluid status assessment.

### 2.4. Laboratory Tests

Complete blood counts were performed with a Beckman Coulter DXH device (Beckman Coulter, Inc., Brea, CA, USA) and biochemical tests were performed with a Beckman Coulter AU5800 analyzer (Beckman Coulter, Inc., Brea, CA, USA). N-terminal pro B-type natriuretic peptide (NT-proBNP) levels were determined with the Elecsys proBNP II test kit using a Roche Diagnostics Cobas E411 device (Roche Diagnostics International Ltd., Rotkreuz, Switzerland).

### 2.5. Statistical Analysis

All statistical analyses were performed using SPSS (Statistical Package for the Social Sciences) version 22.0 (IBM Corp., Armonk, NY, USA). Descriptive statistics are presented as mean ± standard deviation, median (minimum-maximum), frequency and percentage.

For normally distributed continuous variables, we applied paired samples *t*-test, as it is appropriate for comparing means between two related measurements. For non-normally distributed continuous variables, we used the Wilcoxon signed-rank test to compare paired medians. For categorical variables, we employed the Chi-square test to evaluate changes in proportions between baseline and month 3. A value of *p* < 0.05 was considered statistically significant in all tests.

## 3. Results

### 3.1. Patient Characteristics

The mean age of the 77 patients included in the study was 56.18 ± 12.15 years and the age range was 23–87 years. 59.7% of the participants were female (n = 46) and 40.3% were male (n = 31).

The most prevalent malignancies identified in the study were lung cancer (35%) and breast cancer (32.4%). Hypertension (19.5%) and diabetes mellitus (18.2%) were the most prevalent comorbid diseases.

### 3.2. Clinical and Laboratory Findings

The BMI was 27.21 ± 4.58 kg/m^2^ at the beginning of chemotherapy and 26.68 ± 4.64 kg/m^2^ at month 3; the change was not statistically significant (*p* = 0.056).

Significant changes were observed in serum albumin levels, total protein, potassium, magnesium and chlorine values by the third month. In particular, the proportion of patients with albumin levels below 40 g/L decreased from 59.7% at baseline to 41.9% in the third month (*p* = 0.037). No significant difference was found in the sodium level (*p* = 0.131).

An analysis was conducted for the inflammatory biomarker ratios obtained prior to chemotherapy and at the conclusion of the third month. The median value of the platelet/albumin ratio was determined to be 12.4 (5.4–14.9) at baseline and 11.45 (6.3–14.8) at the third month. This decrease was found to be statistically significant (*p* = 0.012). In a similar manner, the neutrophil/albumin ratio was 149.91 (32.54–2130.14) at baseline and 87.49 (11.9–325.43) at the third month, therefore having significantly decreased (*p* = 0.001). However, the neutrophil/lymphocyte ratio was 213.85 (5.07–1034) at baseline and 195.92 (79.47–2040) in the third month, with no significant difference observed between the groups (*p* = 0.814). A subsequent analysis of serum ferritin levels revealed that while the median baseline value was 54.5 (5.3–1198.9) ng/mL, it underwent a significant increase to 109.7 (6.5–762.3) ng/mL in the third month (*p* = 0.001).

A substantial decline was evident in the following parameters: hemoglobin, haematocrit, WBC, neutrophil, and platelet counts. A significant increase in ferritin levels was observed (*p* = 0.001). A substantial increase was identified in MCV (*p* = 0.001). A significant increase was observed in total cholesterol, HDL, and triglyceride levels. A significant decrease in systolic blood pressure was observed (*p* = 0.019).

The data obtained were analyzed statistically, and the significance levels are presented in Table 1.

Biochemical parameters (Table 2) showed significant improvements in serum albumin levels and reductions in total protein, potassium, magnesium, and BUN. Total bilirubin levels increased slightly but significantly. Lipid profile changes included increases in total cholesterol, HDL, and triglycerides. No significant changes were observed in sodium, renal function (creatinine, GFR), or most liver enzymes.

### 3.3. BCM Findings

A comparison of body composition measurements obtained at the onset of chemotherapy and at the third month of treatment revealed a median initial Qualification value of 92% (52–98) and a third-month value of 95% (75–99). This increase was found to be statistically significant (*p* = 0.001). The median ECW value was recorded as 15 (9–27.5) L at baseline and 15.5 (9.3–22.7) L at the third month. The difference between these values was not found to be statistically significant (*p* = 0.058). The median ICW (intracellular fluid) levels at baseline were found to be 15.95 (10.3–43.2) L, and these levels decreased to 15.6 (10.2–26.5) L by the third month. A subsequent analysis revealed that there was no significant difference between the groups (*p* = 0.069). Conversely, the E/I ratio (extracellular/intracellular fluid ratio) exhibited a significant increase in the third month (*p* = 0.001), signifying an augmentation in extracellular fluid.

Subsequent to the analysis of muscle and adipose tissue parameters, it was found that there was no statistically significant difference between the third month and baseline values of LTI (lean tissue index), FTI (fat tissue index), FAT (total fat mass), LTM (lean tissue mass), ATM (adipose tissue mass) and BCM (body cell mass) measurements (*p* > 0.05 for all).

The overhydration (OH) rate was 61% (47/77) at the onset of chemotherapy and increased to 79% (49/62) at the third month. While the number of normovolemic individuals was limited to only four, the number of normovolemic patients reached 13 in the third month, and no hypovolemic individuals were observed. A substantial rise in the proportion of hypervolaemic patients was observed in the third month. Of the eight patients who exited, six had overhydration and two had hypovolaemia. Baseline overhydration was more frequent among patients who died compared with survivors (62.5% vs. 30.0%), although the difference did not reach statistical significance due to the small sample size (*p* = 0.08). No significant association was observed between electrolyte disturbances and mortality.

Patients lost to follow-up by month 3 (n = 15) were excluded from paired comparisons, and no imputation was performed for missing data.

The median baseline OH (EC-EB) was 0.45 (interquartile range: −12.6–18.7) L, and the median 3rd month OH (EC-EB) was 0.51 (interquartile range: −1.1–11.1) L. A significant difference was found between the groups in terms of OH values (*p* = 0.001). The third-month OH value was higher than the baseline OH value.

When the distribution of overhydration difference according to gender was analyzed, the median value was 0.65 L (−1.5–2.4) in females and 1.3 L (−6.4–9.9) in males, and no significant difference was observed between the groups (*p* = 0.132). When evaluated according to chemotherapy regimen, the difference in OH was 1.25 L (−6.4–9.9) in patients treated with cisplatin or carboplatin and 0.65 L (−1.1–2.9) in patients treated with other agents. The difference was not statistically significant (*p* = 0.325). The disparity in mean oxygen levels (OH) among patients with distinct tumor subtypes was examined, revealing a mean difference of 0.75 L (0–2.9) in cases of lymphoma and 0.75 L (−6.4–9.9) in patients without lymphoma. Subsequent analysis revealed no statistically significant difference between these groups (*p* = 0.807) (Table 3).

An evaluation of the correlations between overhydration difference and several clinical parameters yielded a significant, negative, and low-level correlation between the OH difference and the baseline Qualification Value (*p* = 0.002). Conversely, a significant positive yet low correlation was observed between the third month Qualification value and OH difference (*p* = 0.017). No statistically significant correlation was observed between the OH difference and the baseline KxT/V value (*p* = 0.727). A significant negative moderate correlation was identified between the OH difference and the baseline E/I ratio (*p* = 0.001). Furthermore, a significant positive and moderate correlation was observed between the OH difference and the BNP difference (*p* = 0.001). A significant, positive, low-level correlation was also identified between OH difference and baseline calcium level (*p* = 0.016). Conversely, no substantial correlation was identified between baseline and third-month OH levels and systolic and diastolic blood pressure, glucose, sodium, total bilirubin, direct bilirubin, ALP, AST, ALT, GGT, LDH, potassium, chlorine, magnesium, phosphorus, total cholesterol, triglyceride, HDL cholesterol, LDL cholesterol and ferritin levels (*p* > 0.05 for all, Spearman’s Rho Correlation Test used) (Table 4).

In subgroup analysis according to baseline serum sodium level, the median OH difference was 0.7 L (−4.8–3.2) in patients with <135 mEq/L and 0.8 L (−6.4–9.9) in patients with ≥135 mEq/L and there was no significant difference between them (*p* = 0.918). Similarly, in the analysis performed according to the baseline albumin level, the median OH difference was calculated as 0.8 L (−6.4–9.9) in patients <40 g/L and 0.75 L (−1.1–3) in patients ≥ 40 g/L and no statistically significant difference was found (*p* = 0.782).

## 4. Discussion

The incidence and mortality rates of cancer are increasing rapidly worldwide [11]. The aim of this study was to evaluate fluid status and electrolyte changes in patients with malignant disease at the beginning of treatment and after three months. A clinical evaluation was also performed at the time of body cell mass (BCM) measurement. There was a statistically significant decrease in hemoglobin and hematocrit values after three months, compared to the baseline, as well as an increase in the overhydration value, which may have been dilutional. Chemotherapy-related bone marrow toxicity is another possibility. The decrease in white blood cell and neutrophil counts, as well as the decrease in platelet values without a change in lymphocyte values, may have been due to an improvement in the initial inflammation or chemotherapy-related bone marrow toxicity. A significant increase in the MCV value at month three (although these values are within the normal range) may have been related to the improvement in initial inflammation or a deficiency of folic acid and vitamin B12 due to chemotherapy. In the third month, there may have been a relationship between increased overhydration and MCV; however, there was no increase in the ICW value or hyponatremia, though there was an increase in the E/I ratio.

OH increased in the third month of chemotherapy (*p* < 0.001). This may have been related with chemotherapy and the recommendations for standard fluid intake and excessive fluid intake in the follow-ups, indicating an increase in the total body fluid amount. The fluid deficiency we detected at baseline was not detected in the third month.

Cancer patients are generally at risk of developing cachexia as a result of progressive weight loss, loss of muscle and adipose tissue, impaired immune function, decreased food intake, systemic inflammation and abnormal metabolism [12,13]. Many studies have reported that muscle wasting is a strong predictor of the development of chemotherapy toxicity [14,15].

During the treatment and follow-up of our patients, we detected fluid overhydration in the third month. However, this did not adversely affect renal, cardiac, electrolyte, or liver function during the three-month follow-up period. Along with the overhydration, there was a slight increase in BNP values without hyponatremia; however, this increase was not statistically significant. However, there was a significant correlation between the difference in overhydration and the difference in BNP. Due to an insignificant decrease in ICW and an increase in ECW without a change in BMI, LTI, FTI, FAT, LTM, or ATM, it can be concluded that the patients did not develop sarcopenia within the three-month period.

In our study, the increase in OH frequency and slight elevation in BNP levels during chemotherapy, when evaluated in conjunction with the positive and significant correlation between the two variables, suggests that excessive fluid overload may contribute to cardiac stress. The fact that the increase in BNP is related to changes in fluid balance rather than being tumor-related is clinically important, especially in oncology patients with limited cardiac reserve. This situation suggests that early detection of excessive hydration in patients receiving routine fluid therapy during chemotherapy may contribute to the prevention of possible cardiac complications. Additionally, considering that OH may mask muscle mass loss relative to body weight, it was concluded that objective body composition measurements such as BCM could be a valuable tool both in preventing excessive hydration and in predicting mortality risk.

The prevalence of fluid electrolyte and acid-base disorders in cancer patients has been reported to be 58%. This rate is much higher than the rates seen in the general population, in patients admitted to the emergency department for any reason (13.7%) and in the elderly (22%). In a study, lower body surface area, presence of comorbidities such as hypertension, diabetes mellitus, renal failure, anemia, hypoalbuminemia, history of surgery and chemotherapy were found to be major risk factors for electrolyte and acid-base disturbances in cancer patients [16].

Cancer is one of the most common causes of hyponatremia in hospitalized patients. In a prospective observational study, the rate of hyponatremia was reported as 14% [17]. As is the case with the general population, lower sodium levels were found to be associated with a prolonged duration of hospitalization and an elevated 90-day mortality rate [18,19]. In the present study, hyponatraemia was identified as the most prevalent electrolyte imbalance at the commencement of treatment. Tas Et Al. reported that serum sodium concentration had no effect on survival in patients with small cell lung cancer, and therefore concluded that the presence and severity of hyponatraemia had no effect on the outcome of these patients [20].

Hypercalcaemia is a prevalent electrolyte disorder observed in cancer patients. Hypercalcaemia has been observed in 20–30% of cancer patients during the course of the disease [21]. Hypercalcaemia in cancer patients is generally indicative of a poor prognosis.

The decrease in BUN value in the third month of our patients, despite improvement in nutrition or steroid use during chemotherapy, may be related to clinical improvement or malnutrition. It is hypothesized that the absence of change in creatinine value may serve to reduce the likelihood of renal failure. However, it should be noted that malnutrition is a prevalent issue among cancer patients [22].

The prevalence of malnutrition in adult cancer patients ranges from 20% to 87%, contingent on the stage, type, or treatment of cancer [1,2]. Sarcopenia is a condition that invariably worsens with age. Consequently, it is imperative to detect and prevent sarcopenia and malnutrition at the onset of cancer treatment [23,24].

NT-proBNP and troponin T have been identified as valuable markers in the prognosis of oncological diseases, not only in terms of cardiac damage during chemotherapy, but also in terms of prognosis and prolonging the survival of cancer patients [25].

The presence of fluid deficiency or excess in patients was directly correlated with mortality. The presence of pathological findings in fat and muscle mass, as indicated by body composition analysis, is a significant component of a comprehensive clinical evaluation. In a multicentre study excess weight and obesity in 73% of patients, and body composition disorder and decreased muscle mass in the same group. Consequently, the increase in adipose tissue may obscure the decrease in muscle mass [26].

### Limitations

This study has several limitations. First, its single-center design and modest sample size limit generalizability. The three-month observation period may be insufficient to detect long-term outcomes, such as mortality or sarcopenia. The absence of a control group (e.g., non-cancer or non-chemotherapy patients) limits our ability to distinguish chemotherapy-specific changes from other factors.

## 5. Conclusions

At the 3-month mark, signs of inflammation showed improvement in the surviving patients, while serum BNP levels and OH levels increased. It has been established that the presence of hypohypervolemia at the commencement of chemotherapy treatment is associated with an elevated mortality rate. Consequently, the evaluation of body composition by objective methods, such as BCM, to predict mortality and to avoid OH, may facilitate patient management. However, further research is required, involving a larger number of patients and long-term follow-up.

## Figures and Tables

**Table 1:** Hematological and blood pressure changes between baseline and month 3.

Parameter	Baseline	Month 3	*p*-Value
WBC (10^3^/µL)	8	5.7	<0.001
Neutrophil (10^3^/µL)	5.5	3.6	<0.001
Hemoglobin (g/dL)	12.4	11.45	<0.001
Hematocrit (%)	36.9	34.25	<0.001
MCV (fL)	84	87.45	<0.001
Platelet (10^3^/µL)	282.7	252.6	0.032
Ferritin (ng/mL)	54.5	109.7	<0.001
Systolic BP (mmHg)	124.0	118.2	0.019

Abbreviations: WBC, white blood cells; MCV, mean corpuscular volume; PDW, platelet distribution width; BNP, B-type natriuretic peptide.

**Table 2:** Biochemical and Electrolyte Parameters: Baseline Vs. Month 3.

Parameter	Baseline	Month 3	*p*-Value
Albumin < 40 g/L, n (%)	46 (59.7%)	26 (41.9%)	0.037
BUN (mg/dL)	13.8	12.6	0.042
Total Protein (g/L)	69.1	65.86	0.006
Potassium (mEq/L)	4.38	4.21	0.016
Chloride (mEq/L)	103	104	0.004
Magnesium (mg/dL)	1.98	1.87	0.015
Calcium (mg/dL)	9.22	9.4	0.047
Total Bilirubin (mg/dL)	0.41	0.515	0.006
Total Cholesterol (mg/dL)	180.28	200.09	<0.001
HDL Cholesterol (mg/dL)	46.86	51.53	0.009
Triglycerides (mg/dL)	128	158	<0.001

Abbreviations: BUN, Blood urea nitrogen; GFR, Glomerular filtration rate; ALP, Alkaline phosphatase; AST, Aspartate aminotransferase; ALT, Alanine aminotransferase; GGT, Gamma-glutamyl transferase; LDH, Lactate dehydrogenase; HDL, High-density lipoprotein; LDL, Low-density lipoprotein.

**Table 3:** BCM Parameters: Comparison Between Baseline and Month 3.

Parameter	Baseline	Month 3	*p*-Value
QUALIFICATION (%), Median (min–max)	92 (52–98)	95 (75–99)	0.001
ECW (L), Median (min–max)	15 (9–27.5)	15.5 (9.3–22.7)	0.058
ICW (L), Median (min–max)	15.95 (10.3–43.2)	15.6 (10.2–26.5)	0.069
E/I Ratio, Median (min–max)	0.905 (0.34–1.3)	0.96 (0.75–1.25)	0.001
LTI (kg/m^2^), Mean ± SD	11.91 ± 2.65	11.59 ± 2.59	0.153
FTI (kg/m^2^), Mean ± SD	15.01 ± 6.1	14.41 ± 5.74	0.063
FAT (kg), Mean ± SD	29.29 ± 10.53	28.34 ± 10.34	0.074
LTM (kg), Median (min–max)	29.3 (13.6–62.1)	29.9 (17.5–58.4)	0.187
ATM (kg), Mean ± SD	39.86 ± 14.33	38.56 ± 14.07	0.071
BCM (kg), Median (min–max)	15.4 (4.3–37.4)	15.5 (6.7–34.7)	0.159
Overhydration (L), Median (min–max)	0.45 (−12.6–18.7)	0.515 (−1.1–11.1)	0.001

(ECW: Extracellular Fluid, ICW: Intracellular Fluid, E/I: Extracellular fluid/Intracellular fluid ratio, LTI: Lean Tissue Index, LTM: Lean Tissue Mass, FTI: Fat Tissue Index, FAT: Fat, ATM: Adipose Tissue Mass, BCM: Body Cell Mass).

**Table 4:** Correlation of Overhydration Difference with Selected Clinical Parameters.

Parameter	Correlation Coefficient (r)	*p*-Value
Qualification (baseline)	−0.380	0.002
Qualification (Month 3)	0.305	0.017
E/I Ratio	−0.489	0.001
Calcium	0.306	0.016
BNP Difference	0.550	0.001

(E/I: Extracellular fluid/Intracellular fluid ratio, BNP: B-type natriuretic peptide).

## Data Availability

Data are available on request from the authors.

## Abbreviations

The following abbreviations are used in this manuscript:

ALP	Alkaline Phosphatase
ALT	Alanine Aminotransferase
AST	Aspartate Aminotransferase
ATM	Adipose Tissue Mass
BCM	Body Composition Monitor
Bcm	Body Cell Mass
BIS	Bioimpedance Spectroscopy
BMI/VKİ	Body Mass Index
BNP	B-type Natriuretic Peptide
BUN	Blood Urea Nitrogen
EB	Maximum Value
ECOG	Eastern Cooperative Oncology Group
ECW/ES	Extracellular Water
EK	Minimum Value
E/I	Extracellular to Intracellular Water Ratio
FAT	Fat Mass
FMT	Fluid Management Tool
FTI	Fat Tissue Index
FTM	Fat Tissue Mass
GFR	Glomerular Filtration Rate
GGT	Gamma-Glutamyl Transferase
GLOBOCAN	Global Cancer Statistics
HDL	High-Density Lipoprotein
IARC	International Agency for Research on Cancer
ICW/IS	Intracellular Water
KKY	Congestive Heart Failure
KOAH	Chronic Obstructive Pulmonary Disease
LDH	Lactate Dehydrogenase
LDL	Low-Density Lipoprotein
LTI	Lean Tissue Index
LTM	Lean Tissue Mass
MCV	Mean Corpuscular Volume
OH	Overhydration
PC	Personal Computer
PDW	Platelet Distribution Width
PS	Performance Status
PTHrp	Parathyroid Hormone-related Peptide
SPSS	Statistical Package for the Social Sciences
TBW	Total Body Water
TNM	Tumor, Node, Metastasis Classification
TVS	Total Body Fluid
WBC	White Blood Cell Count
